# Simulation Study on Coil of Biomass Carbonization Kettle

**DOI:** 10.3390/ma15062152

**Published:** 2022-03-15

**Authors:** Zuoran Xie, Lei Jiang, Zhibo Cen, Hao Zhang, Bayang Zhang, Jue Zhu

**Affiliations:** 1 Key Laboratory of Impact and Safety Engineering, Ningbo University, Ministry of Education, 818 Fenghua Road, Jiangbei District, Ningbo 315211, China; 1911081014@nbu.edu.cn (Z.X.); 1911081102@nbu.edu.cn (H.Z.); 2111031139@nbu.edu.cn (B.Z.); 2 Ningbo Special Equipment Inspection and Research Institute, 1588 Jiangnan Road, Yinzhou District, Ningbo 315211, China; x3195286769@126.com; 3 Ningbo Metrology and Testing Institute, 1588 Jiangnan Road, Ningbo National Hi-Tech Zone, Ningbo 315211, China; c327311@126.com

**Keywords:** numerical simulation, temperature field, stress field, heat treatment, tensile test

## Abstract

The damage and failure of coiling tube in biomass carbonization kettle due to the long-term operation was discussed. According to the actual structure of the carbonization kettle, a three-dimensional model was established, and the temperature field was simulated based on the given design parameters. The results show that the temperature distribution is stable during 440~450 °C, and the relative error with the actual temperature 449.2 °C, which is about 0.78%. The tensile specimens made of 20G steel, the common material of the coil, was placed in a tubular furnace, and the real heat treatment conditions were simulated with mixed gas and water vapor. After that, the uniaxial tensile test was carried out on MTS testing machine. The obtained physical parameters such as yield strength and elastic modulus were substituted into the numerical temperature field model to obtain the stress field model. The simulation results indicate that during the initial ventilation of the equipment, the coil compressed with the maximum stress of 8.3 MPa at the bending point of the second and fourth laps and partial failure was prone to occur, which is consistent with the actual coil failure result.

## 1. Introduction

Because traditional fossil energy will release a lot of harmful substances such as CO_2_, SO_x_, NO_x_, and dust in the process of utilization, aggravating the deterioration of the environment, and its reserves are decreasing day by day. In the Energy Production and Consumption Revolution Strategy (2016–2030) of the National Development and Reform Commission in China, it is pointed out that the reform of energy structure should be comprehensively arranged, the consumption of coal and other primary energy should be reduced, and the development of clean energy should be promoted [1]. However, China, as a traditional agricultural country, has vast biomass energy reserves. If the efficient mining and utilization of crop surplus value are realized, it will bring vast environmental and economic benefits [2]. Biomass carbonization is a technology that rationally uses the surplus value of crops. It makes use of internal or external heat sources to generate volatiles from biomass pyrolysis and obtain biomass solid carbon, so as to reduce the use of traditional coal.

**Figure 1:**
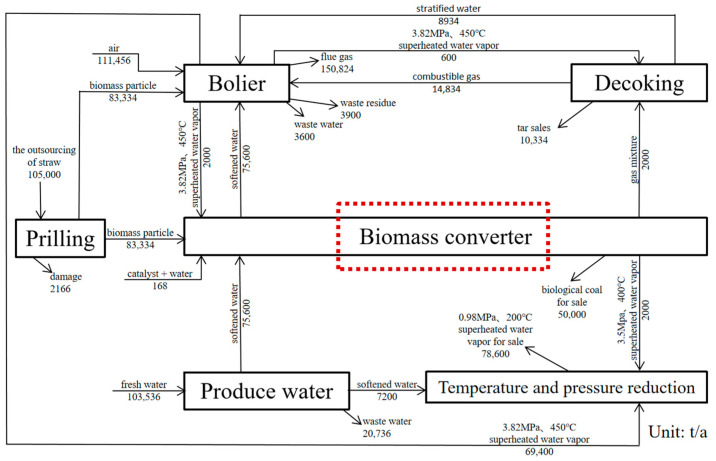
Production process design parameters of annual output of 50,000 tons of biochar.

In view of the problems of low carbonization output, low yield of traditional biomass carbonization kettle, and uneven quality of carbon products [3,4], the project team made a breakthrough on the preparation of biomass molding particles in “Research on Key Technologies and Equipment for the Preparation of Low-Energy Consumption Biomass Molding Fuel”. The proprietary technology for preparing biomass solid carbon [5] by one-step drying-catalysis-carbonization method of indirect heating of biomass pellets with external heat source is proposed. The production process is shown in Figure 1.

Biomass converter (carbonization kettle) is the core equipment of this project. On the one hand, the localization process of biomass carbonization equipment in China has been accelerating; on the other hand, serious scaling, high local temperature, ion erosion, and other phenomena in the coil of biomass carbonization kettle have led to continuous reports of coil equipment damage [6,7,8]. Therefore, it is very important to study the damage and failure mechanism of the coiling tube in the actual working condition of the carbonization kettle.

Temperature is a key index of mechanical property degradation of coil for long-term operation of carbonization kettle [9], while temperature field simulation for the integrity of coil is rarely reported. The simplification of biomass pyrolysis process is a difficulty in temperature field simulation of the whole carbonization kettle according to the actual working conditions. At present, the calculation of heat absorption required by pyrolysis generally adopts the method of assuming constant biomass heat capacity and constant pyrolysis reaction effect [10], but it is difficult to obtain accurate results through this calculation method. Through the analysis of synchronous thermal analyzer (STA), another solution idea can be obtained. Relevant scholars analyzed the thermal physical property curves of various biomass in STA-449C [11] and found that biomass required very little added heat after reaching the pyrolysis temperature. Initially, biomass needs to absorb heat from the outside to break the internal chemical bonds. When the chemical bonds are broken, the heat released by the pyrolysis reaction and repolymerization reaction of the internal functional groups of biomass is enough to maintain the increase of the pyrolysis temperature of biomass and the heat loss of the reactor to the environment, that is, there is no need to obtain energy from the outside [12]. Based on the analysis results of STA by scholars, it can be assumed that all the heat required for pyrolysis carbonization in the carbonization zone is used for biomass heating, thus simplifying the thermal field simulation.

20G steel, as a common material of biomass carbonation kettle coil, has been studied by many scholars. Specimens mass loss method, surface morphology microscopic analysis, and artificial neural network are used to measure the degradation of material properties [13,14,15]; however, are rarely seen in systematic mechanical material tests. In this paper, the gas phase of N_2_, CO_2_, O_2_, and water vapor is used for heat treatment to simulate real corrosion conditions. The material parameters obtained from the tensile test of 20G steel after heat treatment are substituted into the coil temperature field to obtain the coil stress field. The stress concentration of the coil is consistent with the actual damage, which provides theoretical and numerical explanations for the damage of the coil.

## 2. Simulation of Temperature Field

By setting the boundary conditions at the entrance of the carbonization kettle, the temperature distribution in each area of the coil is predicted by heat transfer analysis. The uniformity of the temperature field in the reactor is an important indicator affecting the carbonization quality of biomass particles [16], so the analysis of the temperature field inside the reactor is crucial to verify whether the functional design of the equipment is reasonable.

### 2.1. Modeling and Simplification Assumption

As the computational fluid dynamics analysis software, Fluent software can be used to simulate the fluid mechanics problems in complex mechanisms concerned with the biomass carbonization, such as chemical reaction, combustion, multiphase flow, etc. [17].

**Figure 2:**
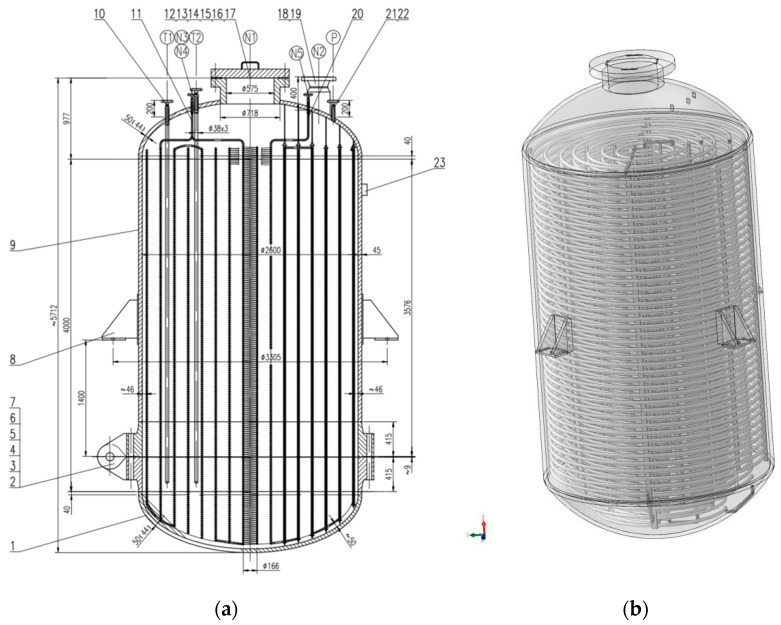
Reactor design diagram. (**a**) Reactor section view; (**b**) 3D model of the reactor.

Biomass converter (carbonization kettle) is the core equipment of this project, and its structure is shown in Figure 2. It is equipped with two entrances, one of which is a spare port, and the overall pipeline is connected by the upper part and the lower part. Temperature and humidity control device and catalyst addition device are evenly set inside the reactor to strengthen heat and mass transfer process and achieve uniform and efficient carbonization; material inlet and outlet adopts flange structure of large diameter, with automatic locking device, to realize automatic feeding and discharging and large-scale production.

As a fixed-bed reactor, the whole heat transfer model is as follows: superheated saturated steam enters the coil through the heat exchanger to form forced convection heat transfer, conducts heat conduction through the wall of the pipe, and finally conducts heat conduction and chemical reaction with the biomass formed particles. Therefore, the heat transfer model of biomass carbonization process can be divided into three parts: the liquid–solid heat exchange between internal heating steam and pipeline; solid–solid heat conduction between pipes and biomass particles; and the chemical reactions that occur during biomass pyrolysis and carbonization [18], the whole process is closely coupled together.

In order to reasonably simplify the model, it is assumed that the biomass materials are relatively static, and the small movement in the carbonization process is not considered. Biomass spherical particles will not occur rupture, wear, and other phenomena; the gas involved in the simulation process is incompressible; all the heat required for pyrolysis and carbonization in the carbonization zone is used for biomass heating [19], these are the simplification of the heat transfer model for the former three parts.

### 2.2. Principle and Material Parameters

The basic principle of ANSYS thermal analysis is to divide the processing object into a finite number of elements (each element contains a number of nodes). Under given boundary conditions and initial conditions, the heat balance equation at each node is solved according to the principle of energy conservation. From this, the temperature values of each node can be calculated and other related quantities can be solved.

The basic mathematical model of temperature field numerical simulation is three-dimensional steady-state partial differential equation of thermal conductivity, Equation (1) [20].

(1) $$\frac{\partial }{\partial x}\left(k_{x}\frac{\partial T}{\partial x}\right)+\frac{\partial }{\partial y}\left(k_{y}\frac{\partial T}{\partial y}\right)+\frac{\partial }{\partial z}\left(k_{z}\frac{\partial T}{\partial z}\right)+\rho \;Q=0$$

where, x, y, z, and are rectangular coordinates; k is thermal conductivity; ρ is material density; Q is the material heat source density; T is the temperature.

In this paper, the third type of boundary conditions, namely, the temperature of the fluid in contact with the boundary and the convective heat transfer coefficient between the boundary surface and the fluid, are used to calculate the heat flux according to Newton cooling formula, Equation (2).

(2) $$q=h(T_{w}-T_{f})$$

where, $T_{w}$ and $T_{f}$ are wall surface and fluid temperature, respectively; h is the convective heat transfer coefficient.

The mathematical model of thermal stress field takes the elastic body as isotropic body. When the temperature of the three-dimensional elastic body changes, it bears the stress caused by the mutual constraints between other objects or internal parts, which can be expressed as, Equation (3).

(3) $$\sigma_{ij}=2G\varepsilon_{ij}+k\varepsilon_{kk}\delta_{ij}-\frac{\alpha E\Delta T}{1-2\nu }\delta_{ij}$$

where, G is shear elastic modulus, ν is Poisson’s ratio, E is tension-compression elastic modulus, δ is Kronecker symbol, α is linear thermal expansion coefficient.

The thermal physical parameters of 20G steel were obtained according to ANSYS software, as shown in the following Table 1.

In the ANAYA software, the Finite Volume Method is used to discrete and solve the existing model. Considering the temperature and pressure as boundary conditions, opening the energy equation and virtual thickness, the standard K−ε model and standard wall function were used as turbulence models. Using the second order upwind format, the simple algorithm is used to solve the problem. The discrete values of each point are approximated by iterative method. In the residuals setting, the energy residuals are of order 10^−7^; the continuity equations and momentum equations are of order 10^−3^.

### 2.3. Model Preprocessing

**Figure 3:**
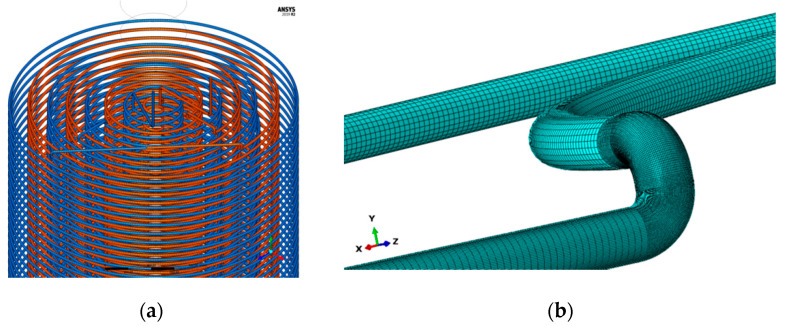
Modeling of coils. (**a**) Reactor coil connection geometry; (**b**) Mesh generation.

In Solidworks modeling, there are eight layers of coils from the inside to the outside, and the overall pipeline is formed by connecting the coils from the upper and the lower, as shown in Figure 3a. The coil cage and other internal devices (temperature and humidity control device and catalyst adding device) are modeled and simplified.

In this article, the hexahedron grid is adopted as the basic element, and the grid size of the main part is 6 mm. In order to avoid the error of stress concentration calculation, the elbow grid size is refined to 1 mm, and the C3D8R grid type is adopted, as shown in the following Figure 3b. Compared with tetrahedral mesh, hexahedral mesh has the following advantages: less quantity, shorter calculation time, and smaller discrete error. The hexahedral mesh direction can better cater to the flow field direction, such as the boundary layer, the calculation is easier to convergence.

**Figure 4:**
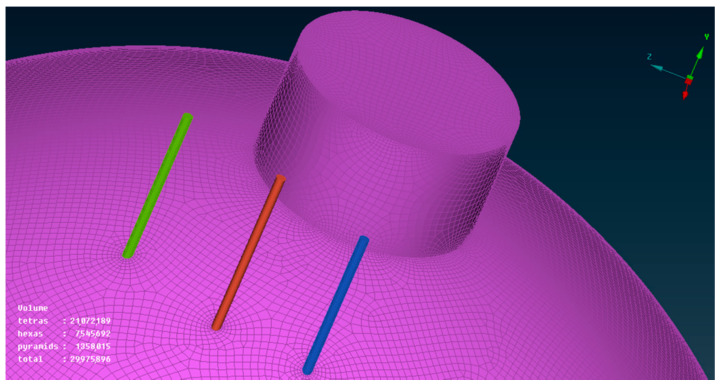
Overall model grid diagram.

In grid division, considering that the focus of the whole model is the distribution of temperature field, there is little need to capture or pay attention to the inner wall of the elbow and the secondary flow of water vapor. According to this idea, there is no need to set expansion layer on the coil grid. A virtual thickness was given when taking the solution setting, and then material attributes were given. In this way, a structured grid of coils and fluid fields can be obtained, which greatly saves computer memory and shortens the solution time. As shown in Figure 4, one can see that the total amount of the simplified grid is also close to 30 million.

### 2.4. Model Check Analysis

According to the actual thermocouple feedback data, the measured constant temperature of coil is 446.2 °C. The sensitivity of grid was tested by changing the size of grid cells, and the results are shown in the following Table 2. When the grid size is 1 mm, the simulation result is precise enough.

**Figure 5:**
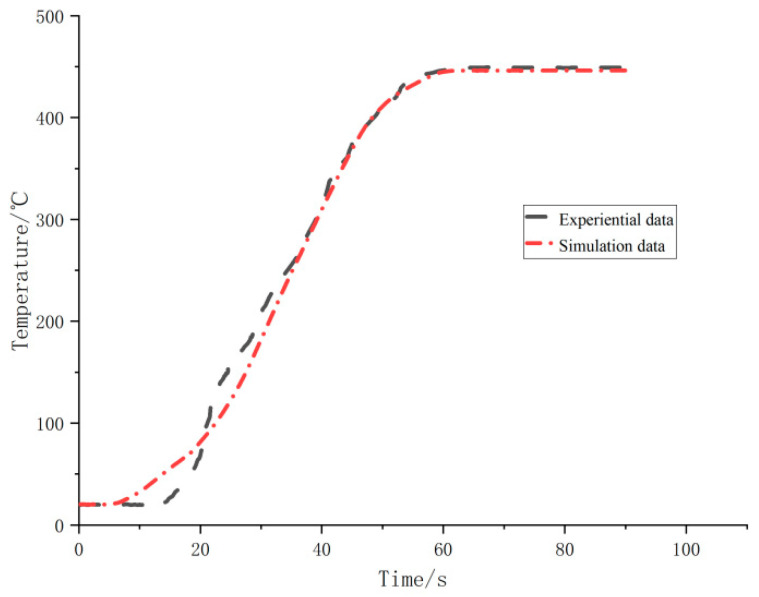
Temperature contrast diagram.

**Figure 6:**
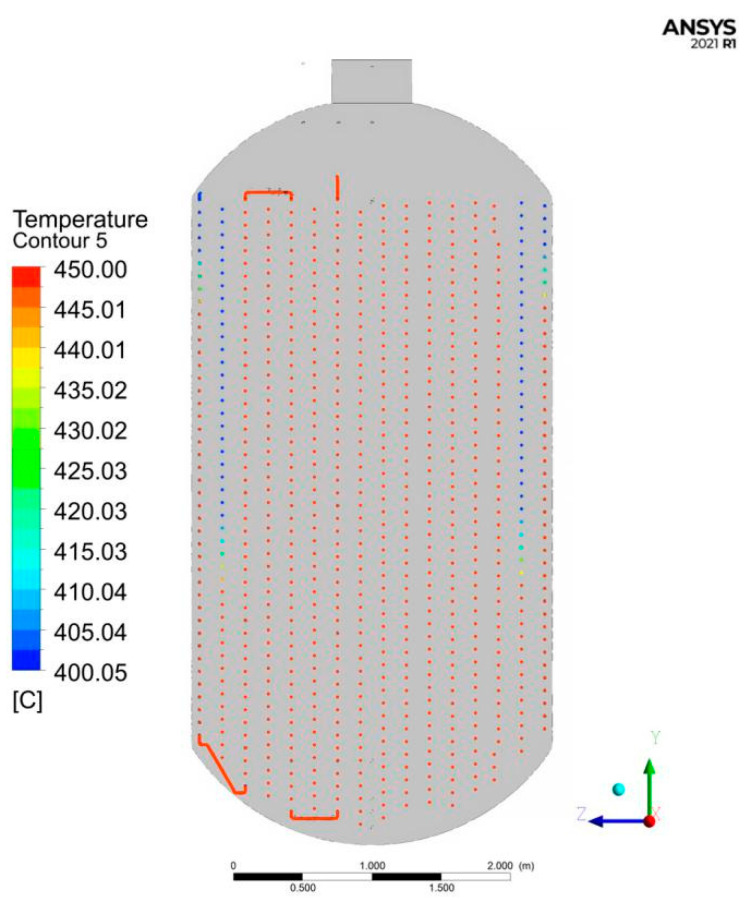
Y-Z plane temperature nephogram of coils.

As shown in Figure 5, the thermocouple temperature sensor feedback experiential data of the inner coil of biomass coil are shown in the black line below, and the simulation data are shown in the red line below. It can be seen that the relative error between the simulated stable temperature (445.7 °C) and the measured stable temperature (449.2 °C) is 0.78%. As can be seen from the temperature field nephogram of coils in the Y–Z plane in Figure 6, the temperature of water vapor is the lowest on the seventh coil from inside to outside. Additionally, the temperature of most coils is between 440 and 450 °C. The internal core temperature is stable, so this simulation can meet the requirements of the uniformity of temperature field distribution in the kettle.

## 3. Temperature Field Simulation

**Figure 7:**
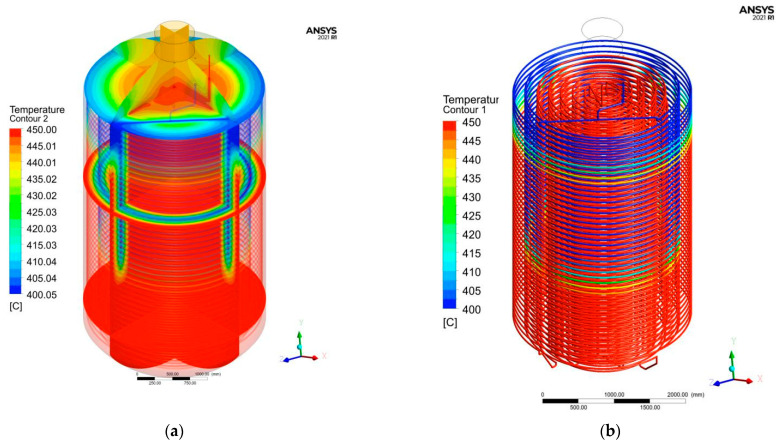
Simulation results of temperature field. (**a**) Nephogram of temperature distribution in carbonization kettle; (**b**) Temperature distribution in Coils.

According to the actual working conditions, the temperature of water vapor heat source at the input port is 450 °C and the pressure is 5.4 MPa. The simplified model and solution process established by simulation and obtained the temperature field results in the carbonization kettle, as shown in Figure 7.

Overall, the temperature in the carbonization kettle is high below and low above, and high inside and low outside. In the area near the outlet of the outer coil, temperature will have certain attenuation, the outlet temperature is lower than that of the inlet air, so near the outlet will be a heat exchange with the surrounding material, and temperature must be lower than the temperature of the core. It can be adjusted through controlling appropriate landfill height of biomass material to control the uniformity of temperature field.

For obtaining the mechanical property of 20G steel used in the simulation, the tensile tests after heat treatment were conducted.

## 4. Tensile Tests after Heat Treatment

### 4.1. Heat Treatment

20G steel has a certain strength under medium and high temperature, low carbon content, good plasticity and toughness, and good cold and hot forming and welding performance. It is the most widely used pressure vessel special steel in China [21]. The tensile specimens were designed according to GB 228.1-2021 ‘Tensile Test of Metallic Materials—Part 1: Test Method at Room temperature’ [22].

**Figure 8:**
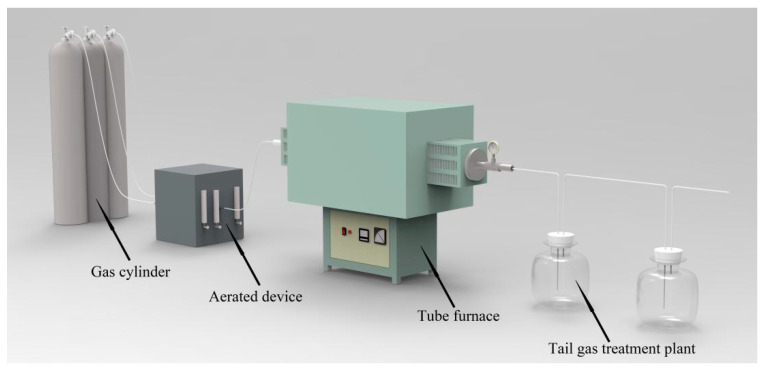
Schematic diagram of heat treatment under simulated working conditions.

The tensile specimens were placed in SK3-4-10 tubular furnace at 25, 440, 450, 500, and 600 °C for 14 h, respectively, and mixed gas of CO, CO_2_, and H_2_ was introduced to simulate the actual working conditions, with the gas ratio of 3:2:1 [23,24]. The total flow rate was 30 mL/min and an appropriate amount of distilled water was injected to simulate water vapor. A schematic diagram of the device is shown in Figure 8 below.

**Figure 9:**
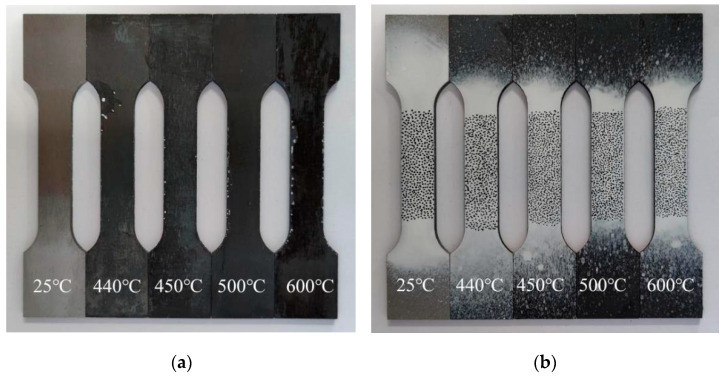
Specimens display diagram. (**a**) The specimen after heat treatment; (**b**) The specimens after spraying speckle.

After the heat treatment under simulated conditions, the tensile specimens was obtained, as shown in Figure 9a. With the increase of heat treatment temperature, the surface of the specimens changes more significantly, and at 600 °C, the surface of the specimens has obvious carbonization peeling phenomenon. Speckles were sprayed on the gauge section of the specimens surface [25], as shown in Figure 9b.

### 4.2. Quasi-Static Tensile Test

**Figure 10:**
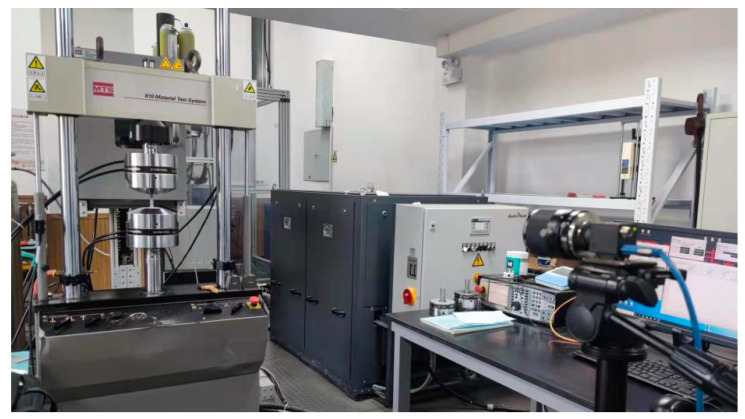
Uniaxial tensile test of specimens.

The uniaxial tensile test was carried out with 10^−3^/s strain rate on model 810 MTS universal testing machine, and a high-speed camera was set up to record the whole process of tensile test. The tensile test process conforms to the national standard GB/T228.1-2010 ‘Tensile test of Metallic Materials—Part 1: Test Method at room temperature’, as shown in Figure 10.

Digital image correlation software was used to analyze the images taken by the high-speed camera to derive the engineering strain of the specimen during tensile process. The engineering stress was obtained by dividing the force recorded on the MTS universal testing machine by the cross-sectional area of the specimens, and then the engineering stress and strain was converted to the real stress and strain according to Equations (4) and (5).

(4) $$\sigma_{T}=\sigma \;\left(1+\varepsilon \right)$$

(5) $$\varepsilon_{T}=ln\;\left(1+\varepsilon \right)$$

In the above formula, $\sigma_{T}$ is the real stress, σ is the engineering stress, $\varepsilon_{T}$ is the real strain, and ε is the engineering strain

**Figure 11:**
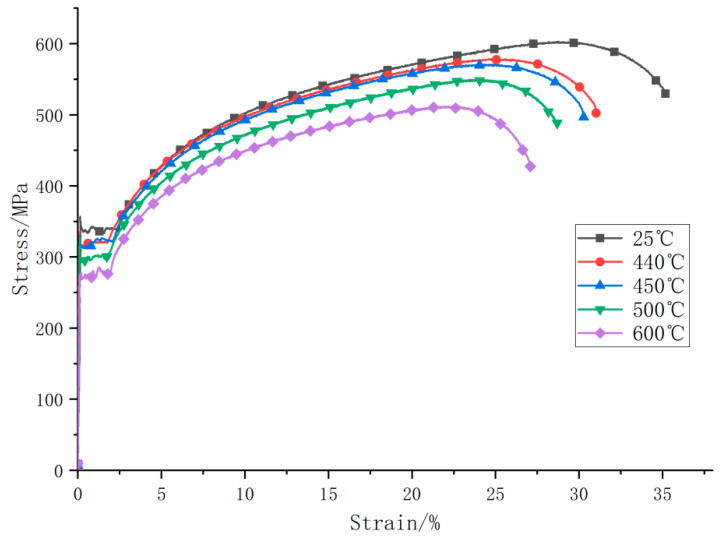
True stress–strain diagram of specimens under uniaxial tension.

The real stress–strain curves of specimens after heat treatment were drawn, as shown in Figure 11 and Table 3 below. It can be concluded that with the increase of the heat treatment temperature, the yield stress and ultimate stress of 20G steel decrease gradually. The higher the working temperature, the more significant the degradation of the mechanical properties of the coil material.

**Figure 12:**
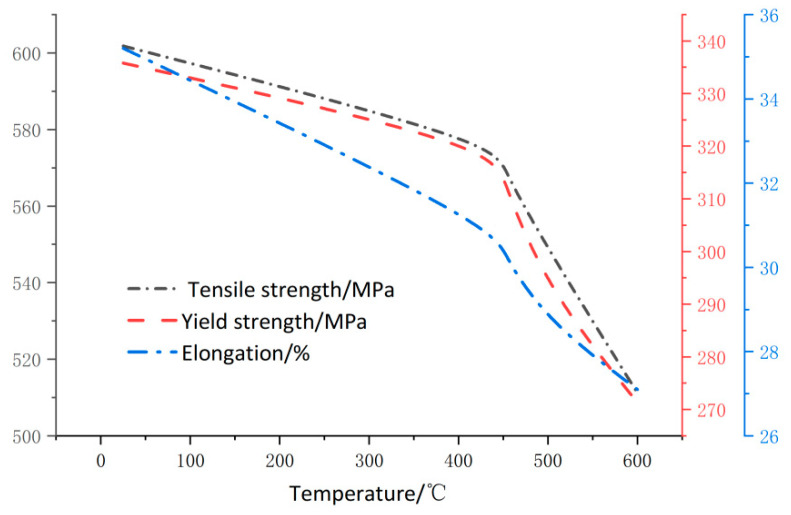
Characteristic strength is correlated.

It can be seen from Figure 12 that the elongation at break is directly proportional to the characteristic strength of the specimen. By comparing the specimens treated at 600 °C with the specimens not treated, it can be seen that the elongation, yield strength, and tensile strength of the specimens are reduced by 23.1, 19.7, and 16.7%, respectively.

**Figure 13:**
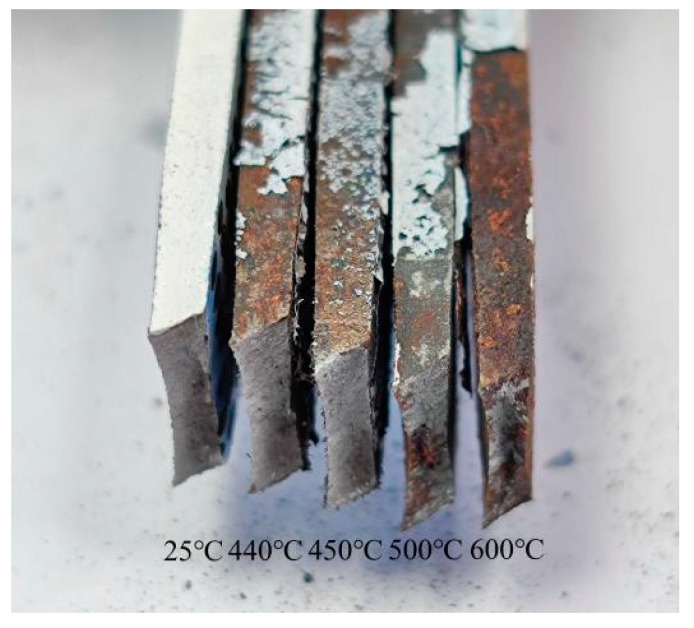
Fracture diagram of tensile specimen.

**Figure 14:**
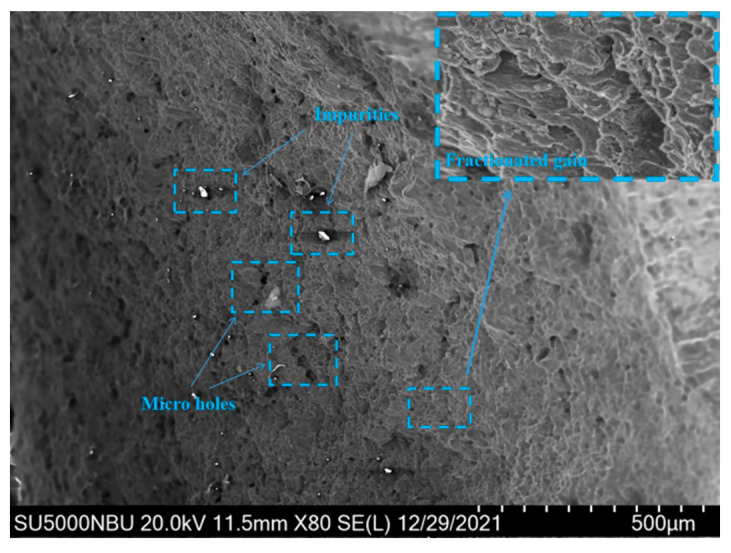
Micrograph of fracture section.

As can be seen from Figure 13, the fracture diagram of the specimens, the fracture surface is at an angle of about 45 °C with the surface of the tensile direction and the smooth surface of the failure section is the fracture formed by shear tearing under tensile. The fracture appears obvious necking phenomenon, which is ductile fracture. It can be seen from the microscopic Figure 14 of the fracture that there are many dimples, which are the traces left on the surface of the fracture after the deformation of the material in the micro-area, nucleation, growth, aggregation, and finally interconnection.

## 5. Stress Field Simulation

**Figure 15:**
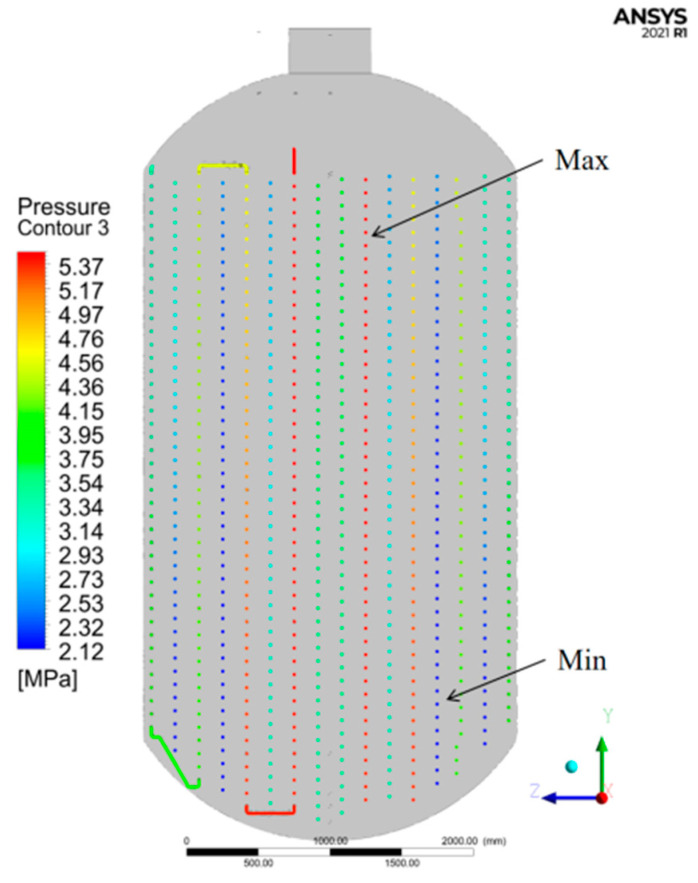
Y-Z plane stress field of coil.

The temperature field above were set as initial condition, the true stress–strain constitutive relation after heat treatment at 450 °C was set as material property of 20G steel, then conduct numerical simulation of structural analysis to obtain the coil stress field. Due to the different connection modes and radiuses of coils in the carbonization kettle, the stress of each coil section on the Y-Z plane is analyzed, as shown in Figure 15. It can be concluded that the minimum stress appears on the fifth coil from inside to outside, and the maximum stress appears on the second coil from inside to outside.

**Figure 16:**
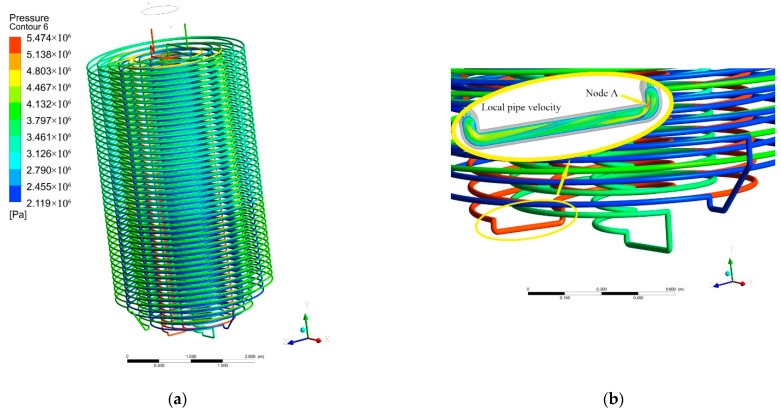
3D stress nephogram of coil. (**a**) Coil’s stress nephogram; (**b**) Local flow diagram of coil.

As can be seen in Figure 16a, regarding the coil’s stress nephogram, it is easy to wear and thin the tube wall when the rapid flow scouring induce the stress concentration at the bending point. In the second and the fourth circles of coils counting from the inside to the outside, the joint stress is about 5.4 MPa, this joint is the most easily prone to failure. In addition, it can be seen from the local airflow diagram in Figure 16b, node A is the most seriously eroded by fluid and is the most easily damaged point at this connection. This is usually the main cause of destruction.

**Figure 17:**
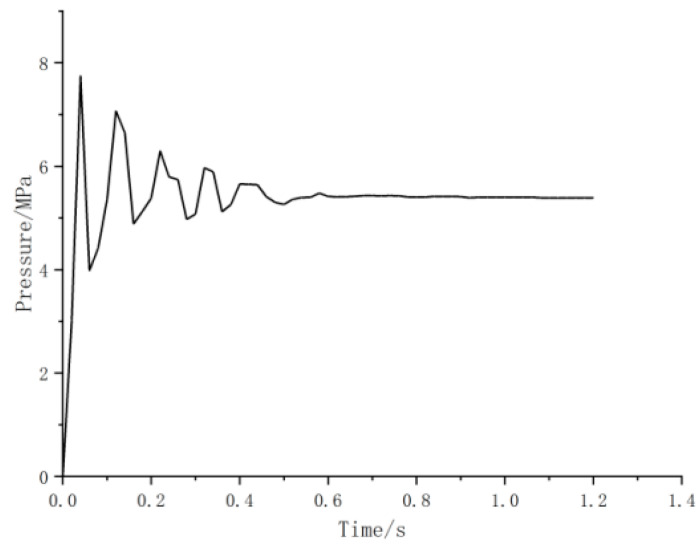
Pressure history curve for node A.

The pressure–time curve of node A was shown in Figure 17. In the initial operation of the equipment, the peak impact pressure is 8.3 MPa, 53.7% higher than that in the constant operation of 5.4 MPa. It can be seen that in the initial ventilation stage, the equipment was affected by the instability of the internal flow field, and the pressure has a sudden peak, which is much higher than the stress in the normal operation condition.

**Figure 18:**
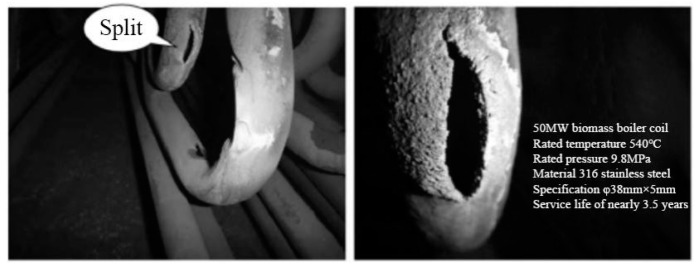
Rupture of coil in actual working condition.

In actual working conditions, most leakage areas of the high temperature superheater are concentrated in the lower part of the tube panel near the bottom elbow, as shown in Figure 18. At the physical level: with the increase of temperature, the mechanical properties of materials degrade accordingly. Moreover, due to chemical corrosion and water vapor erosion, especially at the elbow, the pressure in the local area impacted by airflow is much higher than that in normal operation, which is easy to lead to local damage. The pipe elbow is most likely to be induced by intergranular corrosion cracks in the heat treatment process after bending [26], with a large scouring effect of rapid flow, to result in a local premature failure. The results of numerical simulation are in good agreement with those of real working conditions.

## 6. Conclusions

Based on the actual conditions, the overall three-dimensional modeling of the carbonization kettle were carried out to obtain the overall temperature field of the coil. Taking 20G steel as the object, after heat treatment with mixed gas and steam, which simulated the actual coil working condition, the uniaxial tensile test was carried out. By employing the stress–strain relation of 20G after 450 °C heat treatment and the temperature field as the initial condition, the stress nephogram of coils are obtained. The stress concentration position was consistent with the actual coil damage.

(1) The temperature field distribution of the carbonization kettle shows that the temperature conforms to the concept of uniformity. Most of the coil temperatures are between 440 and 450 °C, and the internal core temperature is stable at 445.7 °C.

(2) Temperature has a significant effect on the degradation of mechanical properties of the 20G steel material. By comparing the specimens treated at 600 °C with the specimens not treated, it can be seen that the elongation, yield strength and tensile strength of the specimens are reduced by 23, 19.7, and 16.7%, respectively.

(3) According to the coil stress field diagram and local flow rate diagram, the stress concentration occurred at the junction of the second and fourth coils, and the peak value was 153.7% at the initial operation of the equipment, which easily led to local damage.

(4) When the equipment is initially started up and running, the inlet should be slowly ventilated and pressurized to avoid coil damage caused by flow field impact.

## Figures and Tables

**Table 1:** Thermophysical property parameter.

Temperature (°C)	Thermal Conductivity (W/(m °C))	Specific Heat Capacity (J/(Kg °C))	Density (g/cm^3^)
20	51.5	460	7.85
100	49.6	476	7.81
200	46.2	489	7.78
300	41.9	502	7.72
400	39.4	519	7.68
500	36.2	530	7.61

**Table 2:** Sensitivity analysis of grid size.

**Grid Cell Size (mm)**	10	3	1	0.5	0.3
**Coil Temperature (°C)**	431.7	440.9	445.7	445.9	446.0

**Table 3:** Physical parameters of 20G steel after heat treatment at 450 °C.

Elasticity Modulus (GPa)	Yield Strength (MPa)	Extension Strength (MPa)	Elongation (%)
257	312	570	30
